# Supplementary material for: Interaction of a viral insulin-like peptide with the IGF-1 receptor produces a natural antagonist
Source: Nat Commun. 2022 Nov 5;13:6700. doi: 10.1038/s41467-022-34391-6 (PMC9637144; doi:10.1038/s41467-022-34391-6)
Supplement: Supplementary file 1 — Supplementary Info File #1 [file 41467_2022_34391_MOESM1_ESM.pdf]

Interaction of a Viral Insulin-Like Peptide with the IGF-1 Receptor Produces A Natural Antagonist

Francois Moreau[1, 10], Nicholas S Kirk[2, 3, 10], Fa Zhang[4], Vasily Gelfanov[4], Edward O List[5], Martina Chrudinová[6], Hari Venugopal[7], Michael C Lawrence[2, 3], Veronica Jimenez[8, 9], Fatima Bosch[8, 9], John J Kopchick[5], Richard D DiMarchi[4], Emrah Altindis[6], C Ronald Kahn[1, *]

1. Section of Integrative Physiology and Metabolism, Joslin Diabetes Center, Harvard Medical School, Boston, MA, USA.

2. WEHI, Parkville, Victoria, Australia

3. Department of Medical Biology, Faculty of Medicine, Dentistry and Health Sciences, University of Melbourne, Parkville, Victoria, Australia

4. Department of Chemistry, Indiana University, Bloomington, Indiana.

5. Edison Biotechnology Institute and Heritage College of Osteopathic Medicine, Ohio University, Athens, OH, USA.

6. Boston College Biology Department, Chestnut Hill, Massachusetts.

7. Ramaciotti Centre for Cryo-Electron Microscopy, Monash University, Clayton, Victoria, Australia.

8. Department of Biochemistry and Molecular Biology, School of Veterinary Medicine and Center of Animal Biotechnology and Gene Therapy, Universitat Autonoma de Barcelona, Bellaterra, Spain.

9. CIBER de Diabetes y Enfermedades Metabólicas Asociadas (CIBERDEM), 28029 Madrid, Spain

10. These authors contributed equally

SUPPLEMENTARY FIGURES

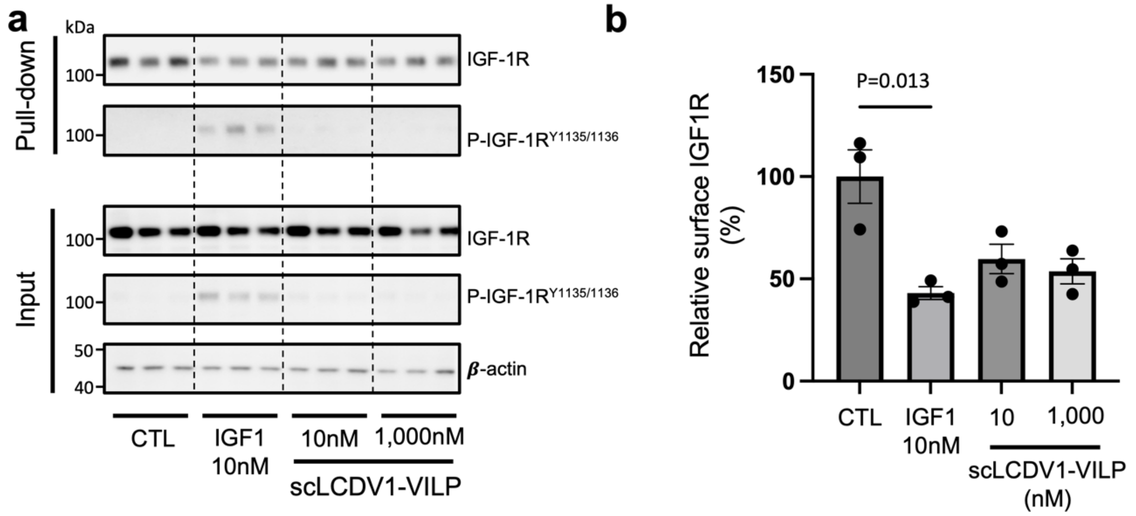

Supplementary Fig. 1: IGF1R internalization following stimulation with IGF1 and scLCDV1-VILP (a) Western blot detection of total and phosphorylated IGF1R and β-actin in cell lysates and biotin labeled membrane proteins fractions. (b) Quantitative analysis of total surface IGF-1R. Data, were normalized to the level of total IGF1R in absence of ligand and expressed as mean ± SEM (Kruskal-Wallis test followed by a Dunn's multiple comparisons test; Graphpad Prism V.9; n=3 independent experiments)

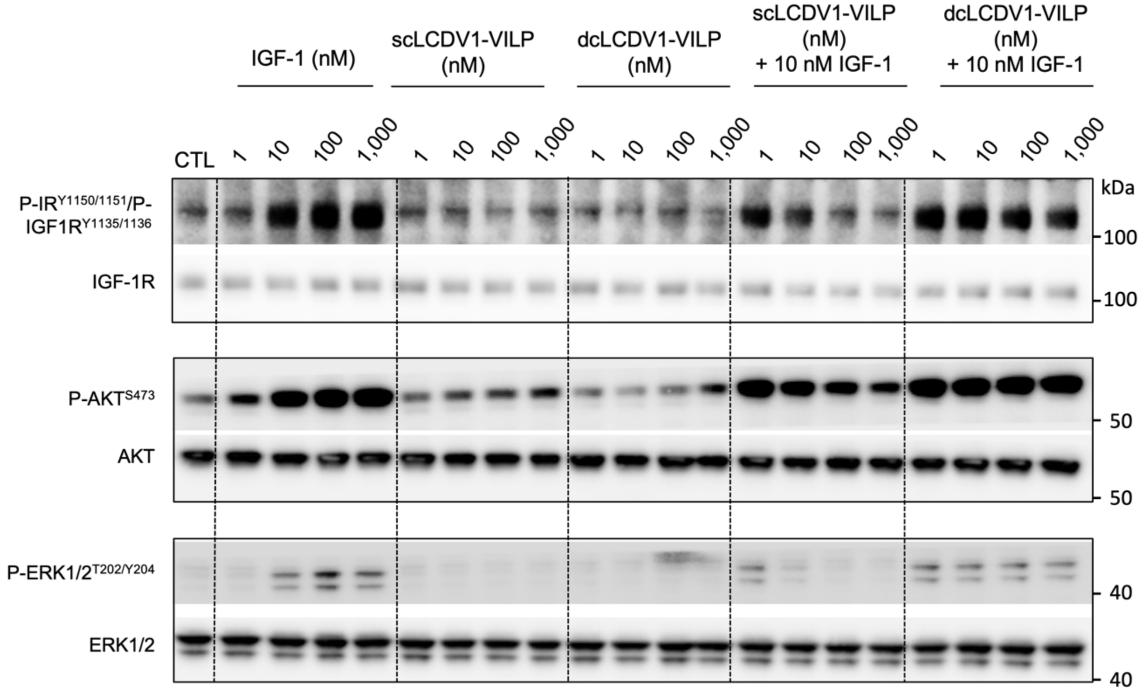

Supplementary Fig. 2: scLCDV1-VILP antagonizes IGF1R signaling in MCF7 cells expressing endogenous levels of IR and IGF1R. Western blot detection of IR/IGF1R, AKT, ERK1 and ERK2 phosphorylation in lysates of MCF7 cells.

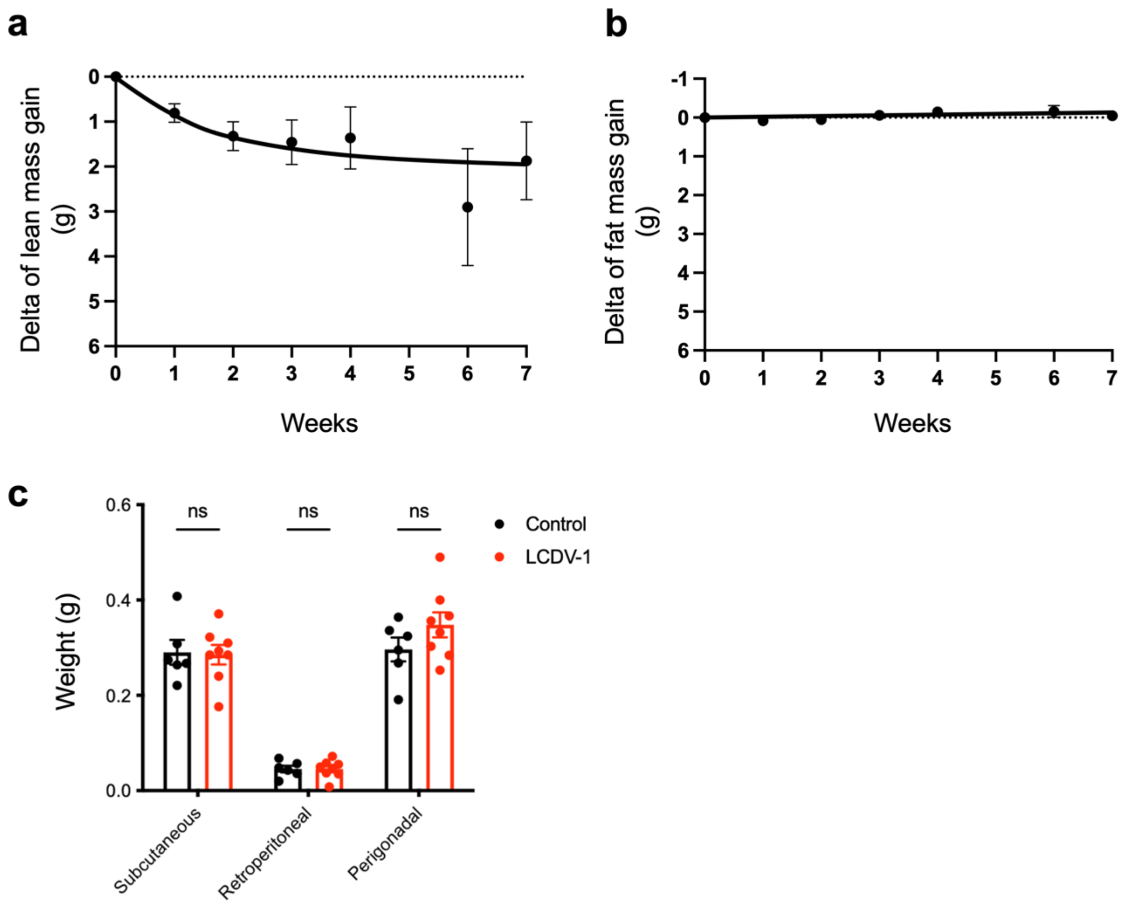

Supplementary Fig. 3: Hepatic LCDV1-VILP expression reduces lean mass without affecting fat mass gain in mice over-secreting bovine growth hormone. Delta of lean mass **(a)** and fat mass **(b)** gain were measured weekly for seven weeks following the AAVs injection. Data were normalized by the basal weight measured at t=0. Data are express as mean ± SEM (AAV null=6 mice, AAV LCDV-1=8 mice). **(c)** Subcutaneous, retroperitoneal and perigonadal fat weight was measured after mice sacrifice. Data are expressed as mean ± SEM (ns, not significant; two-sided Mann-Whitney test; Graphpad Prism V.9; AAV null=6 mice, AAV LCDV-1=8 mice).

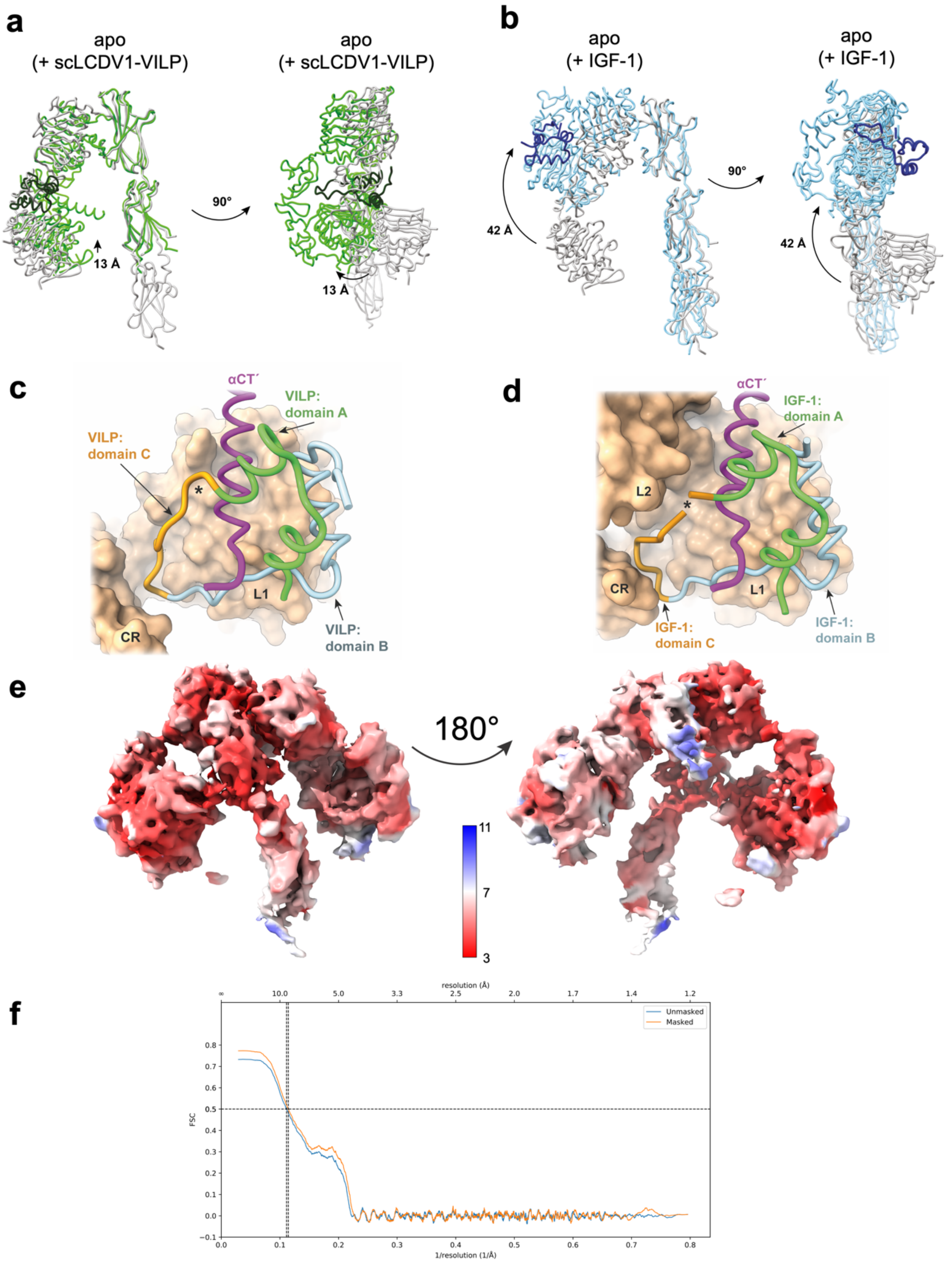
a
apo
(+ scLCDV1-VILP)
apo
(+ scLCDV1-VILP)
90°
13 Å
13 Å
b
apo
(+ IGF-1)
apo
(+ IGF-1)
90°
42 Å
42 Å
c
αCT´
VILP:
domain A
VILP:
domain C
*
L1
CR
VILP:
domain B
d
αCT´
IGF-1:
domain A
L2
*
CR
L1
IGF-1:
domain C
IGF-1:
domain B
e
180°
11
7
3
f
resolution (Å)
Unmasked
Masked
FSC
1/resolution (1/Å)

Supplementary Fig. 4: Conformational change within the primary monomer of IGF1R upon ligand binding. (a) scLCDV1-VILP binding to IGF1R results in a rotation of the L1-CR-L2 module with respect to its downstream FnIII-domain module that effects a *ca* 13 Å displacement of domain L1 with respect to its apo position. A similar change occurs in the alternate αβ monomer (not shown). Within the panel, the apo IGF1R monomer is in grey, the scLCDV1-VILP-bound IGF1R monomer is in green, and the scLCDV1-VILP moiety is in dark green. **(b)** IGF-1 binding to IGF1R results in separate rotations of both IGF-1-bound L1-CR module and of domain L2 with respect to their downstream FnIII-domain module, these rotations effecting a *ca* 42 Å displacement of domain L1 with respect to its apo position. Within the panel, the apo IGF-1R monomer is in grey, IGF-1-bound IGF1R monomer is in cyan, and the IGF-1 moiety is in dark blue. In both (A) and (B), monomers are overlaid based on the common [FnIII-1]+[FnIII-2] module. Comparison of the disposition of the scLCDV1 VILP on the surface of domain L1 **(c)** compared to that of IGF 1 **(d)** on the same surface in its complex with the receptor. The site of difference in the conformation of the C-terminal regions of scLCDV1-VILP and IGF-1 C domains is asterisked. The dashed line represents missing residues that were too ambiguous to be modelled. **(e)** CryoEM density surface colored by local resolution as determined in cryoSPARC. **(f)** Map vs model FSC curve generated during refinement in Phenix.

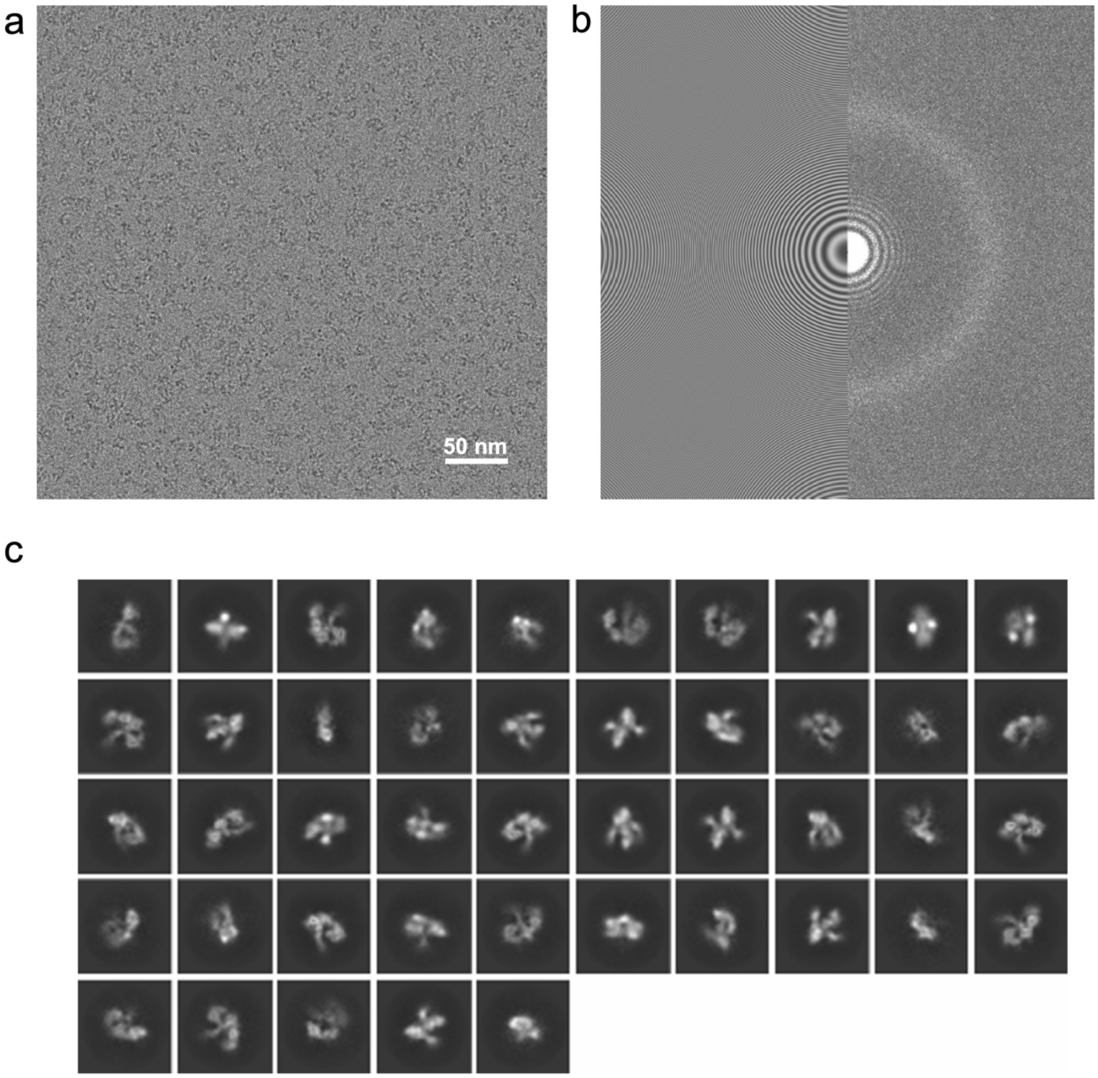

Supplementary Fig. 5: 2D processing of IGF1Rzip + scLCDV1-VILP cryoEM dataset. (**a**) Sample optimal patch-motion corrected micrograph. (**b**) Contrast transfer function associated with (A). (**c**) 2D classes associated with particles retained through 2D classification.

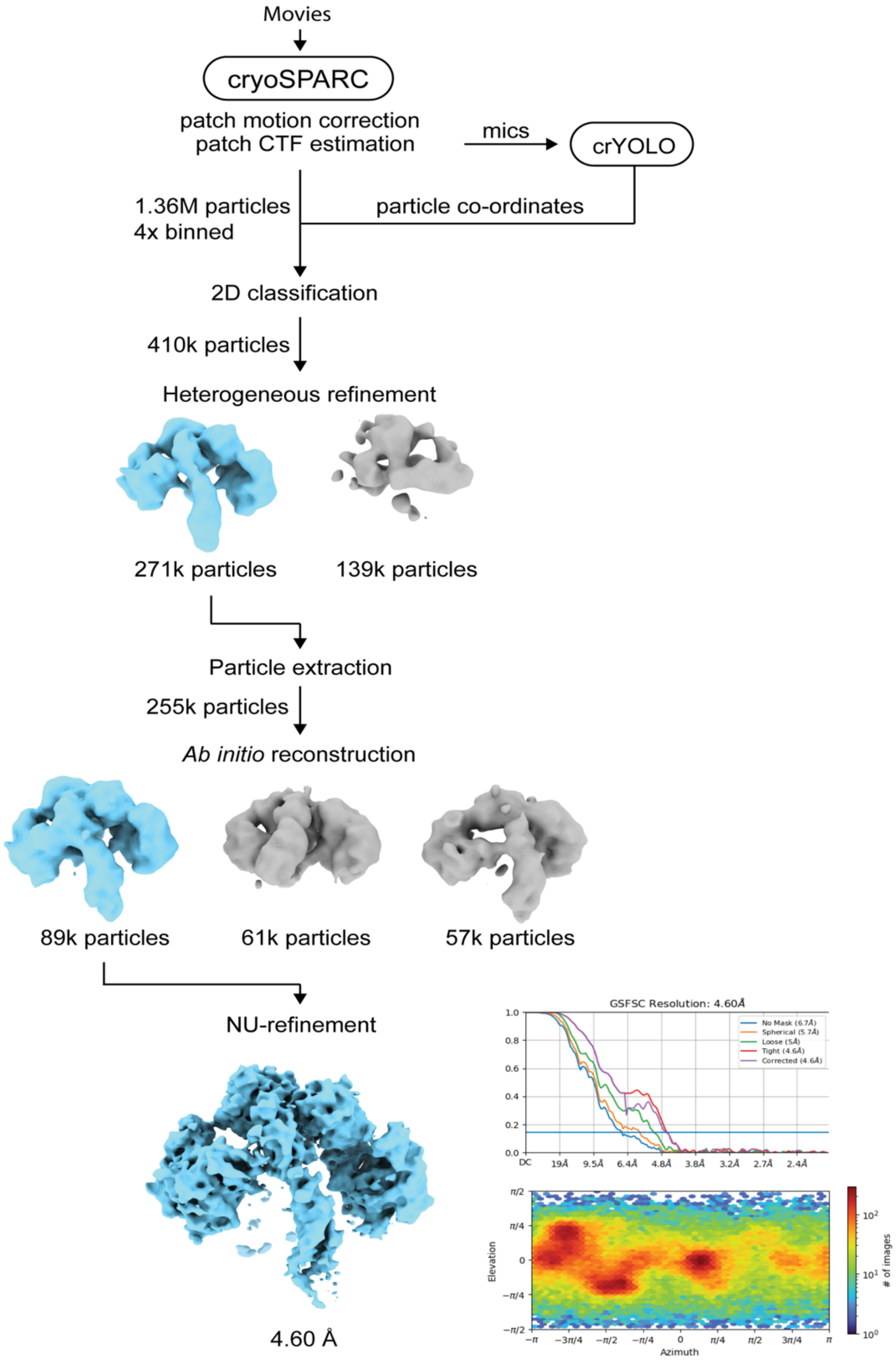

Supplementary Fig. 6: Image processing flowchart for IGF-1Rzip + scLCDV-1 cryoEM dataset.

For explanation and full details, see **Methods** section within the main manuscript.

SUPPLEMENTARY TABLE

Supplementary Table 1: Refinement statistics for cryoEM atomic model

PDB code	7U23
EMDB code	EMD-26306
Composition (#):	
chains	4
atoms (incl. hydrogens)	11688
protein residues	1462
glycan residues	0
Bonds (RMSD):	
length (Å) (# > 4σ)	0.004 (0)
angles (°) (# > 4σ)	0.592 (4)
MolProbity score	2.45
Clash score	14.64
Ramachandran plot (%):	
outliers / allowed / favored	0.35/6.73/92.92
Rotamer outliers (%)	2.67
C^{β} outliers (%)	0.00
Peptide plane (%):	
cis proline / general	2.6/0.0
twisted proline / general	0.0/0.0
C^{a}BLAM outliers (%)	3.02
ADP ($Å^2$):	

iso / aniso (# atoms)	11688
protein (min / max / mean)	44.80/351.65/172.91
glycan (min / max / mean)	n/a
Occupancy (# atoms)	
occ = 1.0 / 0.5 / 0.0	11688/0/0
Map	
Resolution (Å): FSC independent half-maps	4.6
Local resolution range (Å)	2.4 - 48.8
Sharpening B-factor ($Å^2$)	177
Model vs map	
CC_{mask}	0.59
CC_{box}	0.78
CC_{volume}	0.58
CC individual chains:	
IGF-1Rzip (αβ)	0.63
IGF-1Rzip (αβ)′	0.72
scLCDV1-VILP	0.70
scLCDV1-VILP′	0.59
Resolution (Å): FSC, masked map vs model @0.5	4.6, 8.75
