# Supplementary material for: Interaction of a viral insulin-like peptide with the IGF-1 receptor produces a natural antagonist
Source: Nat Commun. 2022 Nov 5;13:6700. doi: 10.1038/s41467-022-34391-6 (PMC9637144; doi:10.1038/s41467-022-34391-6)
Supplement: Supplementary file 2 — Supplementary data 1 [file 41467_2022_34391_MOESM2_ESM.pdf]

>hypothetical protein LCDV1gp068 [Lymphocystis disease virus 1]

Sequence ID: NP_078716.1 Length: 80

Range 1: 17 to 78

Score:126 bits(317), Expect:2e-36,

Method:Compositional matrix adjust.,

Identities:62/62(100%), Positives:62/62(100%), Gaps:0/62(0%)

```
Query  1   ITAEILCSAHLVAALQRVCGNRGVYRPPPTRRRSTRNGTTGIATKCCTTTGCTTDDLEKY  60
           ITAEILCSAHLVAALQRVCGNRGVYRPPPTRRRSTRNGTTGIATKCCTTTGCTTDDLEKY
Sbjct  17  ITAEILCSAHLVAALQRVCGNRGVYRPPPTRRRSTRNGTTGIATKCCTTTGCTTDDLEKY  76

Query  61  CN  62
           CN
Sbjct  77  CN  78
```

>uncharacterized protein LOC110985749 isoform X1 [Acanthaster planci]

Sequence ID: XP_022102687.1 Length: 339

Range 1: 33 to 88

Score:57.8 bits(138), Expect:8e-08,

Method:Compositional matrix adjust.,

Identities:30/57(53%), Positives:36/57(63%), Gaps:1/57(1%)

```
Query  6   LCSAHLVAALQRVCGNRGVYRPPPTRRRSTRNGTTGIATKCCTTTGCTTDDLEKYCN  62
           +C   L+ AL+ VCG+RG Y PPP   R T    TGIAT+CC +  C T  LEKYCN
Sbjct  33  VCGNDLLDALKSVCGDRGFYSPPPGYSRRTPATQTGIATRCCISY-CETSVLEKYCN  88
```

>insulin-like growth factor II [Denticeps clupeoides]

Sequence ID: XP_028824724.1 Length: 168

Range 1: 31 to 88

Score:52.0 bits(123), Expect:4e-06,

Method:Compositional matrix adjust.,

Identities:30/60(50%), Positives:35/60(58%), Gaps:2/60(3%)

```
Query  2    TAEILCSAHLVAALQRVCGNRGVYRPPPTRRRSTRNGTTGIATKCCTTTGCTTDDLEKYC  61
            +AE LC   LV  LQ VCG+RG Y   P  R S R G  GI  +CC  T CT + LE+YC
Sbjct  31   SAETLCGGELVDTLQFVCGDRGFYFSRPN-RLSGRRGYGGIVEECCFRT-CTLELLEQYC  88
```

>PREDICTED: insulin-like growth factor I [Gekko japonicus]

Sequence ID: XP_015278112.1 Length: 164

Range 1: 52 to 110

Score:48.9 bits(115), Expect:6e-05,

Method:Compositional matrix adjust.,

Identities:29/60(48%), Positives:31/60(51%), Gaps:3/60(5%)

```
Query  4     EILCSAHLVAALQRVCGNRGVYRPPPTRRRSTR--NGTTGIATKCCTTTGCTTDDLEKYC  61
             E LC A LV ALQ VCG RG Y   P    S R  N T GI  +CC  + C    LE YC
Sbjct  52    ETLCGAELVDALQFVCGERGFYFSKPAGYGSNRRVNPTKGIVDECCFQS-CDLKRLEMYC  110
```

>insulin-like growth factor I [Ovis aries]

Sequence ID: prf||2001274B Length: 70

Range 1: 3 to 61

Score:47.0 bits(110), Expect:6e-05,

Method:Compositional matrix adjust.,

Identities:29/60(48%), Positives:33/60(55%), Gaps:3/60(5%)

```
Query  4    EILCSAHLVAALQRVCGNRGVYRPPPTRRRST--RNGTTGIATKCCTTTGCTTDDLEKYC  61
            E LC A LV ALQ VCG+RG Y   PT   S+  R   TGI  +CC  + C    LE YC
Sbjct  3    ETLCGAELVDALQFVCGDRGFYFNKPTGYGSSSRRAPQTGIVDECCFRS-CDLRRLEMYC  61
```

Sequence ID: AQY61797.1 Length: 70 >somatomedin C [Homo sapiens]

Sequence ID: prf||0912651A Length: 70

Range 1: 3 to 61

Score:46.6 bits(109), Expect:7e-05,

Method:Compositional matrix adjust.,

Identities:29/60(48%), Positives:33/60(55%), Gaps:3/60(5%)

```
Query  4   EILCSAHLVAALQRVCGNRGVYRPPPTRRRST--RNGTTGIATKCCTTTGCTTDDLEKYC  61
            E LC A LV ALQ VCG+RG Y   PT   S+  R   TGI  +CC  + C    LE YC
Sbjct  3   ETLCGAELVDALQFVCGDRGFYFNKPTGYGSSSRRAPQTGIVDECCFRS-CDLRRLEMYC  61
```

>insulin-like growth factor I [Odocoileus sp.]

Sequence ID: prf||2001274A Length: 70

Range 1: 3 to 61

Score:46.6 bits(109), Expect:9e-05,

Method:Compositional matrix adjust.,

Identities:29/60(48%), Positives:33/60(55%), Gaps:3/60(5%)

```
Query  4   EILCSAHLVAALQRVCGNRGVYRPPPTRRRST--RNGTTGIATKCCTTTGCTTDDLEKYC  61
            E LC A LV ALQ VCG+RG Y   PT   S+  R   TGI  +CC  + C    LE YC
Sbjct  3   ETLCGAELVDALQFVCGDRGFYFNKPTGYGSSSRRAPQTGIVDECCFRS-CDLRRLEMYC  61
```

>RecName: Full=Insulin-like growth factor I; Short=IGF-I; AltName: Full=Somatomedin;

Flags: Precursor [Suncus murinus]

Sequence ID: Q28933.1 Length: 81

>insulin-like growth factor I precursor, partial [Suncus murinus]

Sequence ID: BAA07897.1 Length: 81

Range 1: 7 to 65

Score:46.6 bits(109), Expect:1e-04,

Method:Compositional matrix adjust.,

Identities:29/60(48%), Positives:33/60(55%), Gaps:3/60(5%)

```
Query  4    EILCSAHLVAALQRVCGNRGVYRPPPTRRRST--RNGTTGIATKCCTTTGCTTDDLEKYC  61
            E LC A LV ALQ VCG+RG Y   PT   S+  R   TGI  +CC  + C    LE YC
Sbjct  7    ETLCGAELVDALQFVCGDRGFYFNKPTGYGSSSRRAPQTGIVDECCFRS-CDLRRLEMYC  65
```

>PREDICTED: insulin-like growth factor II isoform X1 [Cyprinus carpio]

Sequence ID: XP_018967675.1 Length: 207

>prepro-insulin-like growth factor-II [Cyprinus carpio]

Sequence ID: AAL25799.1 Length: 207 >hypothetical protein cypCar_00045008 [Cyprinus carpio]

Sequence ID: KTG03935.1 Length: 207

Range 1: 49 to 108

Score:51.2 bits(121), Expect:1e-05,

Method:Composition-based stats.,

Identities:29/61(48%), Positives:35/61(57%), Gaps:2/61(3%)

```
Query  2    TAEILCSAHLVAALQRVCGNRGVYRPPPTRRRSTRNGTT-GIATKCCTTTGCTTDDLEKY  60
            +AE LC   LV ALQ VCG+RG Y   PT R S+R      GI  +CC  + C    LE+Y
Sbjct  49   SAETLCGGELVDALQFVCGDRGFYFSRPTSRLSSRRSQNRGIVEECCFNS-CNLALLEQY  107

Query  61   C  61
            C
Sbjct  108  C  108
```

>insulin-like growth factor II isoform X1 [Clupea harengus]

Sequence ID: XP_031420221.1 Length: 217

Range 1: 54 to 113

Score:51.2 bits(121), Expect:1e-05,

Method:Composition-based stats.,

Identities:29/61(48%), Positives:35/61(57%), Gaps:2/61(3%)

```
Query  2    TAEILCSAHLVAALQRVCGNRGVYRPPPTRRRSTRNGTT-GIATKCCTTTGCTTDDLEKY  60
            +AE LC   LV ALQ VC +RG Y   PT R S R   T GI  +CC  + C  + LE+Y
Sbjct  54   SAETLCGGELVDALQFVCEDRGFYFSRPTGRNSNRRSPTRGIVEECCFRS-CDLNLLEQY  112

Query  61   C  61
            C
Sbjct  113  C  113
```

>insulin-like growth factor II, partial [Chelon labrosus]

Sequence ID: AEI16550.1 Length: 166

Range 1: 7 to 66

Score:50.4 bits(119), Expect:1e-05,

Method:Compositional matrix adjust.,

Identities:29/61(48%), Positives:36/61(59%), Gaps:2/61(3%)

```
Query  2   TAEILCSAHLVAALQRVCGNRGVYRPPPTRR-RSTRNGTTGIATKCCTTTGCTTDDLEKY  60
           +AE LC   LV ALQ VCG+RG Y   PT R  + R  T GI  +CC  + C  + LE+Y
Sbjct  7   SAETLCGGELVDALQFVCGDRGFYFSRPTSRGNNRRTQTRGIVEECCFRS-CDLNLLEQY  65

Query  61  C  61
           C
Sbjct  66  C  66
```
