# Supplementary material for: Interaction of a viral insulin-like peptide with the IGF-1 receptor produces a natural antagonist
Source: Nat Commun. 2022 Nov 5;13:6700. doi: 10.1038/s41467-022-34391-6 (PMC9637144; doi:10.1038/s41467-022-34391-6)
Supplement: Supplementary file 3 — Reporting Summary [file 41467_2022_34391_MOESM3_ESM.pdf]

nature portfolio

Corresponding author(s): C. Ronald Kahn

Last updated by author(s): Sep 4, 2022

Reporting Summary

Nature Portfolio wishes to improve the reproducibility of the work that we publish. This form provides structure for consistency and transparency in reporting. For further information on Nature Portfolio policies, see our Editorial Policies and the Editorial Policy Checklist.

Statistics

For all statistical analyses, confirm that the following items are present in the figure legend, table legend, main text, or Methods section.

n/a	Confirmed	
☐	☒	The exact sample size (n) for each experimental group/condition, given as a discrete number and unit of measurement
☐	☒	A statement on whether measurements were taken from distinct samples or whether the same sample was measured repeatedly
☐	☒	The statistical test(s) used AND whether they are one- or two-sided<br>*Only common tests should be described solely by name; describe more complex techniques in the Methods section.*
☒	☐	A description of all covariates tested
☒	☐	A description of any assumptions or corrections, such as tests of normality and adjustment for multiple comparisons
☐	☒	A full description of the statistical parameters including central tendency (e.g. means) or other basic estimates (e.g. regression coefficient) AND variation (e.g. standard deviation) or associated estimates of uncertainty (e.g. confidence intervals)
☐	☒	For null hypothesis testing, the test statistic (e.g. F, t, r) with confidence intervals, effect sizes, degrees of freedom and P value noted<br>*Give P values as exact values whenever suitable.*
☒	☐	For Bayesian analysis, information on the choice of priors and Markov chain Monte Carlo settings
☒	☐	For hierarchical and complex designs, identification of the appropriate level for tests and full reporting of outcomes
☒	☐	Estimates of effect sizes (e.g. Cohen's d, Pearson's r), indicating how they were calculated

Our web collection on statistics for biologists contains articles on many of the points above.

Software and code

Policy information about availability of computer code

Data collection	Microsoft Excel V16.64.
Data analysis	Graphpad prism (V.9) was used for statistical analysis. ImageJ (v2.3.0/1.53q) was used to quantify protein bands on immunoblots. Multiple sequence comparison by log-expectation was performed using Molecular Evolutionary Genetics Analysis software (MEGA v10.1.8). AlphaFold v2 was used to predict three-dimensional structures and the structures were visualized using ChimeraX v1.3. CryoSPARC v3.2 was used for cryoEM image analysis. Particle coordinates were determined using crYOLO v1.8.0 beta. The model was rigid body fit using Phenix v1.19.2 4158 000. Torsion and Ramachandran restraints was undertaken using Coot v0.9.

For manuscripts utilizing custom algorithms or software that are central to the research but not yet described in published literature, software must be made available to editors and reviewers. We strongly encourage code deposition in a community repository (e.g. GitHub). See the Nature Portfolio guidelines for submitting code & software for further information.

Data

Policy information about availability of data

All manuscripts must include a data availability statement. This statement should provide the following information, where applicable:

- Accession codes, unique identifiers, or web links for publicly available datasets
- A description of any restrictions on data availability
- For clinical datasets or third party data, please ensure that the statement adheres to our policy

The data that support the findings of this study are available upon request. Electon microscopy struture is available on protein data bank (PDB) under the accession ID 7U23. Copies of receptor modules L1 CR, L2, FnIII 1, and FnIII 2 were extracted from PDB entry 5U8R. The Two copies of the extended alpha-CT segment were extracted from PDB entry 6PHY

Field-specific reporting

Please select the one below that is the best fit for your research. If you are not sure, read the appropriate sections before making your selection.

☒ Life sciences ☐ Behavioural & social sciences ☐ Ecological, evolutionary & environmental sciences

For a reference copy of the document with all sections, see nature.com/documents/nr-reporting-summary-flat.pdf

Life sciences study design

All studies must disclose on these points even when the disclosure is negative.

Sample size	Sample size was determined in line with previous study with similar experiments (PMID 29467286, PMID 33220491). Each sample size is detailed in the methods or in the figure legends.
Data exclusions	No data were excluded from the analyses.
Replication	All measure were replicated at least 3 times unless stated otherwise in the figure legends.
Randomization	All cells were randomly assigned to experimental group and mice were randomized by the body weight.
Blinding	The use of two different cell lines (brown preadipocyte expressing either the human IR or IGF1R) and different treatments at various concentration necessitate to not be blind. However everithing was made to performed each measure and analysis in an unbiased manner (for example, the use of an automatic cell counter instead of counting the cells manually to asses the cell growth, Fig 4A and B).

Reporting for specific materials, systems and methods

We require information from authors about some types of materials, experimental systems and methods used in many studies. Here, indicate whether each material, system or method listed is relevant to your study. If you are not sure if a list item applies to your research, read the appropriate section before selecting a response.

Materials & experimental systems

n/a	Involved in the study
☐	☒ Antibodies
☐	☒ Eukaryotic cell lines
☒	☐ Palaeontology and archaeology
☐	☒ Animals and other organisms
☒	☐ Human research participants
☒	☐ Clinical data
☒	☐ Dual use research of concern

Methods

n/a	Involved in the study
☒	☐ ChIP-seq
☒	☐ Flow cytometry
☒	☐ MRI-based neuroimaging

Antibodies

Antibodies used	The following antibodies were used in this study:<br>IRS-1 (monoclonal) (mouse) (catalog 611394) from BD Bioscience (diluted 1:1000)<br>p-IRS-1Y895 (polyclonal) (rabbit) (catalog 3070) from CellSignaling (diluted 1:1000)<br>IR (monoclonal) (catalog 3025) from CellSignaling (diluted 1:1000)<br>p-IRY1150/1151/IGF1RY1135/1136 (monoclonal) (rabbit) (catalog 3024) from CellSignaling (diluted 1:1000)<br>IGF1R (polyclonal) (rabbit) (catalog 3027), from CellSignaling (diluted 1:1000)<br>AKT (monoclonal) (rabbit) (catalog 4685) from CellSignaling (diluted 1:1000)<br>p-AKTSer473 (monoclonal) (rabbit) (catalog 4060) from CellSignaling (diluted 1:1000)<br>ERK1/2 (polyclonal) (rabbit) (catalog 9102) from CellSignaling (diluted 1:1000)<br>p-ERK1/2T202/Y204 (monoclonal) (rabbit) (catalog 4370) from CellSignaling (diluted 1:1000)<br>goat anti-rabbit HRP conjugated (catalog 1706515) from BioRad (diluted 1:1000)<br>sheep anti-mouse HRP conjugated (catalog NA931) from Sigma-Millipore (diluted 1:1000)
Validation	All antibodies used in this study were comercially developed and used in previous studies. Information regarding validation and application can be found on manufacturer's website.<br>IRS-1 (mouse) (catalog 611394) https://www.bdbiosciences.com/content/bdb/paths/generate-tds-document.nz.611394.pdf<br>p-IRS-1Y895 (rabbit) (catalog 3070), https://www.cellsignal.com/datasheet.jsp?productId=3070&images=1<br>IR (rabbit) (catalog 3025) https://www.cellsignal.com/datasheet.jsp?productId=3025&images=1<br>p-IRY1150/1151/IGF1RY1135/1136 (rabbit) (catalog 3024) https://www.cellsignal.com/datasheet.jsp?productId=3024&images=1<br>IGF1R (rabbit) (catalog 3027) https://www.cellsignal.com/datasheet.jsp?productId=3027&images=1<br>AKT (rabbit) (catalog 4685) https://www.cellsignal.com/datasheet.jsp?productId=4685&images=1

p-AKTSer473 (rabbit) (catalog 4060) https://www.cellsignal.com/datasheet.jsp?productId=4060&images=1
ERK1/2 (rabbit) (catalog 9102) https://www.cellsignal.com/datasheet.jsp?productId=9102&images=1
p-ERK1/2T202/Y204 (rabbit) (catalog 4370) https://www.cellsignal.com/datasheet.jsp?productId=4370&images=0
goat anti-rabbit HRP conjugated (catalog 1706515) https://www.bio-rad.com/webroot/web/pdf/lsr/literature/LIT418.pdf
sheep anti-mouse HRP conjugated (catalog NA931) https://www.sigmaaldrich.com/US/en/product/sigma/gena931100ul

Eukaryotic cell lines

Policy information about cell lines

Cell line source(s)	Brown preadipocytes knocked-out for the endogenous insulin and IGF-1 receptors and overexpressing either the human IR or the human IGF-1 receptor were generated at the Joslin Diabetes Center by isolating preadipocytes from newborn IR lox/IGF1R lox and then immotalized using a retrovirus coding for the SV40 T-antigen as descibed in Cai, W., Sakaguchi, M., Kleinridders, A. et al. Domain-dependent effects of insulin and IGF-1 receptors on signalling and gene expression. Nat Commun 8, 14892 (2017). https://doi.org/10.1038/ncomms14892.<br>MCF-7 cells were purchased from Genecopoeia (SL017).
Authentication	These cell lines were authentified in the Dr. Kahn's lab by qPCR and immunoblots.
Mycoplasma contamination	The cell lines were tested negatively for mycoplasma.
Commonly misidentified lines<br>(See ICLAC register)	No commonly misidentified lines were used in this study

Animals and other organisms

Policy information about studies involving animals; ARRIVE guidelines recommended for reporting animal research

Laboratory animals	Mus musculus, C57BL/6J, female, 19 days old. Bovine growth hormone transgenic mice were generated and identified as previously described in (PMID: 15231300). Mice were housed 2-4 per cage and given ad libitum access to water and rodent chow (ProLab RMH 3000; 14% of energy from fat, 60% from carbohydrates, and 26% from protein; PMI Nutrition International, Brentwood, Missouri). The cages were maintained in a temperature (22°C) and humidity-controlled room and exposed to a 14-h light, 10-h dark cycle.
Wild animals	The study did not involve wild animals.
Field-collected samples	the study did not involve samples collected from the field.
Ethics oversight	All procedures were approved by the Ohio University Institutional Animal Care and Use Committee and fully complied with all federal, state, and local policies.

Note that full information on the approval of the study protocol must also be provided in the manuscript.
